# The effect of stromal vascular fraction and Platelet-Rich Plasma combination on basic Fibroblast Growth Factor serum level during anal trauma healing in a Wistar rat model

**DOI:** 10.1016/j.amsu.2022.103375

**Published:** 2022-02-11

**Authors:** Ricko Sadli Sujana, Sulmiati, Nita Mariana, Fonny Josh, Sachraswaty Rachman Laidding, Andi Alfian Zainuddin, Muhammad Faruk

**Affiliations:** a Department of Surgery, Hasanuddin University, Makassar, Indonesia; b Division of Pediatric Surgery, Department of Surgery, Hasanuddin University, Makassar, Indonesia; c Division of Pediatric Surgery, Department of Surgery, Wahidin Sudirohusodo Hospital, Makassar, Indonesia; d Division of Plastic and Reconstructive Surgery, Department of Surgery, Hasanuddin University, Makassar, Indonesia; e Division of Plastic and Reconstructive Surgery, Department of Surgery, Wahidin Sudirohusodo Hospital, Makassar, Indonesia; f Department of Public Health and Community Medicine Science, Hasanuddin University, Makassar, Indonesia

**Keywords:** Basic fibroblast growth factor, Stromal vascular fraction, Platelet-rich plasma, Anal trauma, Wound healing

## Abstract

**Introduction:**

Stromal Vascular Fraction (SVF) and Platelet-Rich Plasma (PRP) application play important roles in the healing process by increasing basic Fibroblast Growth Factor (bFGF) secretion. This research assesses the effect of combined SVF and PRP local injection on bFGF levels, using an anal trauma model in Wistar rats.

**Method:**

Twenty-eight adult Wistar rats were divided into three groups. Groups A and B underwent modified surgical anal trauma and repair; Group A was treated with the SVF and PRP combination local injection, while Group B was treated with only normal saline. Subsequently, we examined bFGF levels in Groups A and B on days 1, 7, and 14. Group C consisted of healthy controls sacrificed on day 0 to obtain baseline data on bFGF levels.

**Results:**

The bFGF levels were higher in Group A than in Group B on every experimental day. The Repeated Measures test shows a significant increase in bFGF levels on day 1 (p = 0.000), day 7 (p = 0.000), and day 14 (p = 0.000). This test also indicates that the local injection combination of SVF and PRP increased bFGF levels by 96.2% compared to the placebo group.

**Conclusion:**

The combination of SVF and PRP can increase bFGF levels during anal trauma healing in the Wistar rat model. Basic FGF is an important factor throughout the anal trauma healing process.

## Highlights

• The combination of SVFs and PRP is effective to increase the concentration of bFGF.
• Administration of SVFS + PRP by injection was better at increasing the expression of bFGF than by placebo.
• The combination of SVFs and PRP increases the level of bFGF in the healing process of anal trauma.

## 1 Introduction

Anorectal trauma cases are rare among both adults and children, due to their anatomy. Anal trauma is more prevalent because of the superficial location of the anus [1]. The prevalence of anorectal injury among children at the Primary Children's Medical Center in Utah was 0.2% in the period of 2003–2012. Forty-three percent of these cases involved the anus, 38% involved the rectum, and 19% involved the anorectal complex [2]. Child anorectal injury is usually caused by accident or sexual abuse [3].

Management of anorectal trauma consists of a combination of evaluation and treatment, focused on the primary survey, secondary survey, the location, and extent of the trauma [4]. Some clinicians apply the principle of 4D (Debridement, proximal Diversion, Drainage, and Distal washout), along with surgery [2,4,5].

Disturbance during the anal trauma healing process may cause fecal incontinence in up to 19% of cases [2]. Fecal incontinence may lead to a 3–10% mortality rate if it is neglected [5]. This fecal incontinence results from anal sphincter injury at the time of trauma or after surgery. Another complication that often arises in cases of anal trauma is anal stenosis, which often occurs due to impaired postoperative healing. Anal stenosis is caused by the formation of non-elastic cicatrical tissue, which narrows the anal lumen [6]. Treatment with stem cells has shown promising outcomes in managing this complication [7].

A general wound healing is a dynamic process with multiple complex phases (inflammation, proliferation, and remodeling); it involves interactions between specific cells and the extracellular matrix, coordinated by growth factors, cytokines, and chemokines [[8], [9], [10]]. The inflammatory phase occurs immediately after injury to prevent infection, lasting 1–2 days in uncomplicated wounds. This process consists of a vascular response (hemostasis) and a cellular response (inflammatory) [8]. This inflammatory process is characterized by the presence of tissue edema [11]. The proliferative phase begins 2–10 days after injury. This process is characterized by the appearance of granulation tissue and the occurrence of angiogenesis [8]. The final phase is remodeling, which begins 2–3 weeks to 2 years post-injury [9]. Under normal conditions, the wound undergoes a wound healing process, leaving avascular scar tissue [12].

The intestinal organ has different healing phases compared to the skin; these are restitution, proliferation, and cell differentiation. These differences have been investigated thoroughly due to the intestine's one-layered columnar epithelial cells and different types of collagen cells; these are characterized by higher collagenase activity, the presence of both aerobic and anaerobic environments, and the need for adequate tissue perfusion during the healing process [13,14].

As mentioned above, the wound healing process requires growth factors. One of the most important growth factors in intestinal wound healing is Fibroblast Growth Factor 2 (FGF-2), which is generally known as basic FGF (bFGF). Basic FGF is prevalent in intestinal FGF receptors (FGFR) [15] and plays a role in intestinal tissue recovery and regeneration [9] during the inflammation and proliferation phases [16].

Stem cell application has shown remarkable efficacy in accelerating the phases of the wound healing process [17]. The stromal vascular fraction (SVF)—isolated from adipose tissue—contains adipose-derived stem cells (ASCs) and limited growth factor. SVF has regeneration and anti-inflammation potential due to its ability to secrete bFGF [18]. Platelet-rich plasma (PRP) exerts its role by stimulating bFGF secretion, which activates fibroblasts and the deposition of new collagen [19]. The combination of SVF and PRP yields a synergistic effect that improves the healing of osteoarthritis [20], rat burn injury [11], and human alopecia [21].

Previously, Sirowanto et al. found that local injection of an SVF and PRP combination within an anal trauma model increased the epidermal growth factor (EGF) level during the healing process [22]. In this study, we aim to determine the effect of an SVF and PRP combination on bFGF levels during anal trauma healing.

## 2 Methods

We performed experimental research using the Wistar rat model with a post-test control group design that included 2 experimental groups and 1 healthy group. The subjects were obtained from and treated in The Animal Laboratory of the Faculty of Medicine, Indonesian Muslim University, in Makassar, Indonesia.

All procedures were conducted with the approval from Hasanuddin University Faculty of Medicine Ethics Committee (recommendation number 412/UN4.6.4.5.31/PP36/2021). This research was conducted ethically according to the ARRIVE (Animal Research: Reporting of In Vivo Experiments) guidelines for animal research [23].

## 3 Population and sample

The experimental subjects were 28 (calculated using Federer's Formula) healthy Wistar rats (*Rattus novergicus*), male, aged 16–24 weeks, and weighing 170–260 g; these were divided into 3 groups. Groups A and B (each consisting of 12 rats) underwent modified anal surgical trauma and repair; group A (the treatment group) was treated with a local injection of combined SVF and PRP after surgery, while, group B (the placebo group) was treated with local injection of normal saline. We subsequently measured the bFGF levels in Groups A and B on days 1, 7, and 14. Group C consisted of 4 healthy rats; they received no trauma and were sacrificed to obtain data on the baseline bFGF level.

## 4 SVF preparation

We adapted the SVF preparation protocol described by Josh et al. [24]. We took adipose tissue from the bilateral inguinal regions of the Wistar rats, excised and minced the tissue, washed it extensively using phosphate-buffered saline (PBS, Gibco-BRL, Grand Island, NY, USA), and then placed it into a new tube. We added 0.15% collagenase solution (Wako Pure Chemical Industries, Ltd., Osaka, Japan) and centrifuged it at 37 °C for 30 min. In the next step, we added equal volumes of Dulbecco's Modified Eagle Media solution (DMEM, Gibco-BRL, Grand Island, NY, USA), 1% antibiotic-antimycotic solution (Gibco-BRL, Grand Island, NY, USA), and 10% fetal bovine serum (FBS, Gibco-BRL, Grand Island, NY, USA) to neutralize the collagenase activity. We centrifuged this suspension at 1500 rpm for 5 min. We took the lower layer (pellet), counted the SVF cells in the Neubauer chamber, and separated 50,000 SVF cells for the final SVF product.

## 5 PRP preparation

We contained the blood sample collected from the donor rats within an EDTA-filled tube. We performed a double centrifuge procedure to obtain a PRP [20,24]. First, we centrifuged the sample at 2400 rpm for 10 min, yielding a 3-layered solution. We took the upper layer (supernatant plasma) and the middle layer (buffy coat) and centrifuged them a second time at 3600 rpm for 15 min, which yielded a 2-layered solution. We took the lower layer (infranatant buffy coat) as the final PRP [24].

## 6 SVF and PRP combination preparation

We combined 50,000 SVF cells with activated PRP (PRP-added 10% CaCl_2_) to obtain a final volume of approximately 0.5 mL.

## 7 Wistar rat anal trauma model

The model used in our research was adapted and modified from Trebol et al. [25] and consists of the following steps:

1. We sedated the rat using ether inhalation.
2. We placed the rat in the supine position and performed the aseptic and antiseptic procedures on the anal and perineal areas.
3. We emptied the rectum using manual massage and inserted a 6-Fr foley catheter as a marker.
4. We performed a vertical anterior perianal incision (10 mm) and identified and dissected the adipose tissue until the submucosal layer was reached, while avoiding injury to the anal mucosal layer (watched carefully for visible catheter). If perforation occurred, we approximated the mucosal layer with interrupted stitches, using absorbable sutures (6-0 RB-1 17 mm 1/2c Taper Coated VICRYL Ethicon).
5. We repaired the submucosal and muscular layer with interrupted stitches, using absorbable sutures as mentioned above.
6. For Group A, we gave an SVF and PRP combination injection (0.5 mL) between the intestinal serous layer and the subcutaneous layer, with a divided dose of 0.25 mL on each side of the surgical wound. In Group B, we injected a total of 0.5 mL of normal saline, using the same approach as in Group A.
7. We closed the skin incision with interrupted stitches, using absorbable sutures as mentioned above; the surgical wound was washed with normal saline.
8. We observed and placed all experimental rats in a cage after they regained consciousness; they were given antibiotic (amoxicillin 50 mg/kg BW/day) and analgetic (paracetamol 10 mg/kg BW/day) treatment for 3 days. The rats had free access to food and water.

## 8 Rat sacrifice and ELISA procedure

The rats were anesthetized using ether inhalation before being sacrificed; we then secured the rats on top of the surgery table. We performed a thoracotomy procedure to obtain a blood sample from the heart apex. The aspirated blood was collected for ELISA examination in HUM-RC.

## 9 BFGF ELISA procedure

We used a bFGF ELISA Kit (My BioSource.com, Rat Fibroblast Growth Factor ELISA Kit, Catalog #MBS260671) with procedure steps as follow:

1. Reagents, samples, and standards were prepared.
2. The prepared samples and standard were added and incubated at 37 °C for 90 min.
3. The samples were washed twice, supplemented with Biotinylated Antibody solution, and incubated at 37 °C for 60 min.
4. The samples were washed 3x, supplemented with the enzyme working solution, and incubated at 37 °C for 30 min.
5. The samples were washed 5x, the Color Reagent solution was added, and the samples were incubated at 37 °C for up to 30 min.
6. Color Reagent C was added.
7. Amicroplate reader was used to measure the OD within 10 min of adding Color Reagent C.
8. The content of the samples being tested was determined.

## 10 Statistic analysis

The numerical experimental data were analyzed using the Repeated Measures test, carried out with IBM SPSS Statistics version 26.0 (IBM SPSS Statistics for Windows, Version 26.0. IBM Corp., Armonk, NY). The results are presented in tables and graphics.

## 11 Result

We used 28 Wistar rats that were consistent with the inclusion criteria; these were divided into Groups A, B, and C. Group A served as the treatment group while group B was the placebo group. Group C included 4 healthy rats; they were not subjected to any trauma or injection but were sacrificed on day 0 to obtain baseline data.

The univariate analysis test shows the mean ± standard deviation of bFGF levels in each group, as seen in Table 1. According to Table 1, the mean level in Group C was 113.14 ± 13.54; this was the baseline mean bFGF level for this research. This result shows that a normal healthy rat has a mean bFGF level of 113.14 ± 13.54 pg. The mean for Group A on day 1 was 148.27 ± 26.51; on day 7, it was 181.00 ± 26.20; on day 14, it was 387.18 ± 31.17. In group A, the mean on each day increased continuously compared to the day before and remained higher than the baseline value. The means in Group B were 96.55 ± 23.01, 114.05 ± 28.98, and 167.5 ± 19.39 on days 1, 7, and 14, respectively. The mean for Group B was lower than the baseline on day 1; it then began to increase above the baseline. Generally, the mean levels in Group A were higher than the mean levels in Group B on every experimental day.

**Table 1:** Basic FGF levels according to univariate analysis.

Data		N	Mean ± SD (pg/ml)	Minimum	Maximum
Day 1	A	4	148.27 ± 26.51	116.73	177.53
	B	4	96.55 ± 23.01	70.36	125.41
Day 7	A	4	181.00 ± 26.20	150.12	206.28
	B	4	114.05 ± 28.98	91.69	155.82
Day 14	A	4	387.18 ± 31.17	341.57	410.49
	B	4	167.5 ± 19.39	149.21	192.26
C		4	113.14 ± 13.54	102.65	131.73

Note: A = treatment group; B = placebo group*;* C = healthy group; N = sample number; SD = standard deviation.

All data were analyzed further to determine their significance. First, we identified the normality and homogeneity of the data distribution. The results of the Shapiro-Wilk Normality Test and Mauchly's Test of Sphericity were p > 0.05 for every group on every experimental day, indicating that the data distribution was normal and homogenous. Furthermore, we chose the Repeated Measures Test to analyze all data because the data were assumed to be matched paired data, where all rats were consistent with the inclusion criteria. The result of the Repeated Measures Test was p = 0.000, with an F value of 148.34 for the comparison within and between groups on the experimental days. This result shows that the mean of Group A was statistically significant within the group itself, as well as compared to Group B on experimental days 1, 7, and 14. The Repeated Measures Test also shows a partial eta squared value of 96.2%, indicating that the local injection of the SVF and PRP combination increased serum bFGF levels by 96.2% compared to the placebo group. The aforementioned description is illustrated in Table 2.

**Table 2:** The Repeated Measures Test within and between groups.

Data		bFGF Mean ± SD (pg/ml)	Nilai p			
			Shapiro-Wilk Normality Test^a^	Mauchly's Test of Sphericity^b^	Repeated Measures Test^c^	Partial Eta Squared
Day 1	A	148.27 ± 26.51	0.923	0.408	0.000 (F = 148.34)	96.2%
	B	96.55 ± 23.01	0.979			
Day 7	A	181.00 ± 26.20	0.553			
	B	114.05 ± 28.98	0.224			
Day 14	A	387.18 ± 31.17	0.144			
	B	167.5 ± 19.39	0.611			

Note: A = treatment group; B = placebo group; SD = standard deviation; a = normal data distribution if p > 0.05; b = homogenous data distribution if p > 0.05; c = significant if p < 0.05; p = probability.

**Fig. 1:**
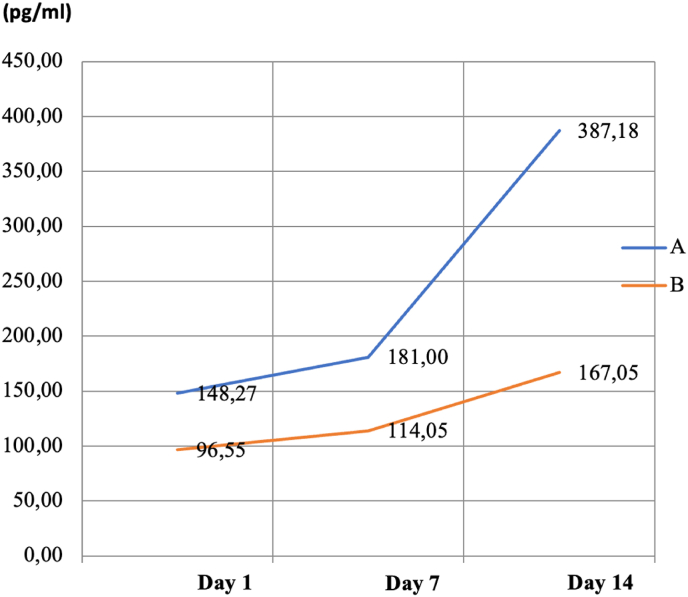
Profile plot of mean bFGF level. Note: A = treatment group; B = placebo group.

A comparison within and between the experimental groups is illustrated in Fig. 1. The mean values in Groups A and B were higher than the day before. All of the mean values in Group A were above those of Group B. The illustration shows that the bFGF levels in both groups had not reached a turning point even 14 days after the experiment.

## 12 Discussion

Basic FGF plays an important role in the wound healing process by promoting the proliferation and migration of fibroblasts [[26], [27], [28]]. Basic FGF levels increase during the healing process with several conditions, such as burn injury, surgical incision, a broken bone, chronic ulcer, and diabetes [29]. Iizuka et al. and Lyra Junior et al. have noted that bFGF works mostly during the intestinal restitution and proliferation phases [13,14]. Mansoub et al. reports that PRP and adipose-derived stem cell (ASC) treatment can increase bFGF expression levels [30]. Research conducted by Rachman et al. and Josh et al. suggests that combined SVF and PRP treatment can boost healing from burn injuries in rats [31,32]. Applying SVF and PRP in combination also improves healing from osteoarthritis in rats, as well as human alopecia [21,33].

At the time of anal trauma, as in Groups A and B, the gastrointestinal wound healing process occurs; this includes restitution, proliferation, and cell differentiation. The process of epithelial restitution begins within minutes to hours after injury and is independent of proliferation. The proliferation of the mucosal epithelium begins in the hours or first several days after the wound, when there is an increase in the enterocyte reserve to close the wound [13]. Lyra Junior et al. have found that the process of restitution occurred from within several minutes to the third day after trauma, while the proliferative process occurred from day 4 to day 14 after trauma [14].

When the restitution phase occurs, the damaged intestinal epithelial cells lose their polarity and immediately repair themselves. The actin cytoskeleton is reorganized under the control of the Rho-GTP pathway to form a new epithelial membrane. Platelets are activated by thrombin to form a fibrin clot that becomes a reservoir for monocytes, neutrophils, and fibroblasts to work. The fibrin clot attracts more neutrophils and monocytes to the wound site. Basic FGF and other cytokines are secreted from the formed fibrin clot. Monocytes differentiate into macrophages, which act as mediators of the proliferative phase. In the proliferative phase, the abundant macrophages stimulate the secretion of bFGF. Macrophages attract and stimulate fibroblasts, and bFGF then induces fibroblast proliferation to form myofibroblasts that synthesize collagen [8,9,13,34,35].

The addition of an SVF and PRP combination accelerates the healing process by producing a synergistic effect. PRP, with its abundant platelets, forms a larger fibrin clot, which then attracts more neutrophils and monocytes to the wound site and secretes more bFGF and other cytokines. PRP, with its fibrin clot, helps the limited growth factors and ASC included in SVF to adhere within the wound site [33]. PRP promotes the proliferation and differentiation of ASC [36,37]. Furthermore, ASCs play a role in proliferation and angiogenesis by acting as an anti-apoptotic and anti-inflammatory agent that promotes epithelialization and neovascularization processes [25,38].

In Group A, the mean bFGF levels increased significantly on all experimental days compared to the mean in Group B. This result differs slightly from those in the study conducted by Mansoub et al. who investigated the effect of PRP and mesenchymal stem cell (MSC) applications on burn injuries in rats; they found that the mean bFGF level continued to increase until day 10 and then decreased until day 14 [30].

Our study results indicate that the SVF and PRP combination injection increased bFGF levels during the research process; this increase was even statistically significant since the first day of the study. In theory, the addition of SVF that already contains growth factors—especially when supported by PRP, which improves SVF performance—would increase growth factors [37]. We assumed that, in addition to natural growth factors from rats, the addition of the external combination of SVF and PRP increased serum bFGF levels directly. In the study by Mansoub et al. the decrease in the treatment group from day 10 resulted from the start of the remodeling process in their research model [30].

The results for Group B show that the mean bFGF level continuously increased over the previous day, but it the mean appeared to be higher than that of Group C on day 14. A study with rat skin incisions by Adil et al. shows similar results, where the mean of bFGF level was significantly higher on day 14 when compared to controls [39]. An intestinal fistulation study by Gao et al. also found similar results [40]. Another study on ulcerative colitis induced with iodoacetamide found decreased bFGF levels in the first 6 h, which then increased until the 10th day of the study [41]. However, these results differ from those of a study on rat burn injury by Mansoub et al. who found an increase in bFGF until the 7th day, followed by a decrease on the 14th day [30].

In Group B, the average bFGF level increased from day to day, although the results were higher than the baseline data on the 14th day. Gao et al. explain that the decrease in early days compared to baseline values resulted from the loss of many growth factors at the time of trauma; these values then began to increase when there was activation of platelets and fibrin, which are sources of growth factors including bFGF. The increase in bFGF levels until day 14 and the fact that it had not reached its turning point in our experiment may stem from the abundance of active fibroblast cells and the major migration of macrophages to the wound site, causing bFGF expression to continue increasing [40]. We assume that our wound model had not reached the remodeling phase. In their study, Mansoub et al. found that the mean bFGF level increased until the 7th day and decreased until the 14th day; they state that it resulted from the beginning of the remodeling phase in their research wound model [30]. In the remodeling phase, fibroblast cells and macrophages begin to disappear as a de-collagenation process is initiated, which results in the decrease of bFGF [9,30].

In this study, the administration of SVF and PRP was proven statistically to increase the mean bFGF level. Local injection of those combinations increased the serum bFGF level by 96.2% compared to the placebo group. Basic FGF plays a very important role in the healing process of anal trauma in Wistar rats. Considering the increase in bFGF levels during the study and the important role of bFGF in wound healing, we believe that the SVF and PRP combination could accelerate the healing of anal trauma wounds or prevent stenosis and other complications in anal trauma healing. Therefore, these hypotheses should be tested by further research.

## 13 Conclusion

This research shows that the addition of SVF and PRP together can increase bFGF levels in the rat model during anal trauma healing. Basic FGF is one of the most important growth factors during the healing process. Therefore, SVF and PRP administered in combination have the potential to elevate growth factor levels during the healing process.

## 14 Provenance and peer review

Not commissioned, externally peer-reviewed.

## Ethical approval

All procedures were conducted with the approval from Hasanuddin University Faculty of Medicine Ethics Committee (recommendation number 412/UN4.6.4.5.31/PP36/2021).

## Sources of funding for your research

No funding or sponsorship.

## Author contribution

Ricko Sadli Sujana, Sulmiati, Nita Mariana, Fonny Josh, and Sachraswaty Rachman Laidding wrote the manuscript and participated in the study design. Ricko Sadli Sujana and Sulmiati drafted and revised the manuscript. Ricko Sadli Sujana and Muhammad Faruk performed anal trauma treatment and surgery. Ricko Sadli Sujana and Andi Alfian Zainuddin performed bioinformatics analyses and revised the manuscript. All authors read and approved the final manuscript.

## Registration of research studies

None.

## Consent

This manuscript does not involve human participants, human data, or human tissue.

## Guarantor

Sulmiati and Fonny Josh.

## Declaration of competing interest

The authors declare that they have no conflict of interests.
